# Passing Topics of the Day

**Published:** 1915-10-02

**Authors:** 


### PASSING TOPICS OF THE DAY.

### Women Members of Boards of Guardians.

Miss Crosbie, Lady Mayoress of the City in 1912, has been appointed a member of the St. Pancras Board of Guardians; Mrs. Irving, the wife of Mr. H. B. Irving, is President of the St. Pancras Infirmary.

### A New Departure Needed at Guy's.

The urgent need of trust funds for providing the poor with artificial teeth was referred to at an inquest recently held by the City Coroner. The case under investigation was that of a man who had the remains of two teeth only—on opposite sides of his mouth—and whose death was caused by suffocation, a piece of meat three inches long having lodged in his larynx. In summing up, Dr. Waldo said that some two years ago he held an inquest in the same district on a man who was practically starved owing to want of teeth, natural or artificial. At that time the Coroner pointed out the numerous ills—mostly preventable—due to the want of teeth, and expressed the hope that some good Samaritan would come along and start a fund to remedy this. As a direct result of a good report of this inquest being read by Mr. Edwin Tate, the Edwin Tate Trust Fund of £5,000 was started at St. Bartholomew's Hospital, and the scheme approved by the Charity Commissioners. Dr. Waldo hopes that the recent case may also lead to the foundation of a similar trust fund at Guy's Hospital, which is still unprovided for in this way. We concur in this wish of the Coroner, and trust that another benefactor will follow the example set by the brother of the founder of the Tate Gallery.

### The "Best" Precautions Against Zeppelin Fires: The Police View.

In view of the possibility of further attacks by hostile aircraft, the Commissioner of Metropolitan Police recently reissued a public warning that no reliance can be placed upon many of the dry powder fire extinguishers which are being so much advertised. Such methods may be very serviceable in the case of small fires, but they can hardly be expected to be effective in controlling fires such as are likely to be caused by bombs, explosive or incendiary. The provision and prompt and intelligent use of water or sand, or of both, in dealing with such outbreaks of fire is, in the opinion of the Commissioner, the best, simplest, and most economical safeguard. Of course there are on the market various forms of chemical liquid fire extinguishers which are extremely serviceable, but no hospital authority should ever purchase any of these appliances without a written guarantee that they comply with the specifications of the Board of Trade, Office of Works, Metropolitan Police, or the approved Fire Prevention Council.

### A Prison as a Bed Factory.

We have already referred to the splendid way in which Canada is co-operating with the Mother Country in making provision for the treatment of the wounded, and we learn this week that the Ontario Government is supplying 1,500 beds, 1,500 mattresses, and 3,000 blankets for use in Canadian hospitals in England. It is interesting to note that the beds are being made at the Guelph Central Prison, which is able to turn out fifty enamel beds per day.

### A Night Patrol?

The Islington Board of Guardians have decided to insure their buildings against air raids, and have further *appointed a night watchman to patrol the grounds for a month on trial.*

### Poor-Law Infirmaries and Aircraft.

In view of the possibility of further raids by hostile aircraft, the St. Pancras Guardians have decided to effect an insurance for £279,940 on their infirmaries and other institutions to cover these risks. The premium is about £270.

### Atmospheric Pollution in Leicester.

The Medical Officer of Health at Leicester has made arrangements for the fixing of an apparatus, issued by the National Society for the Prevention of Atmospheric Pollution, in an open space in the front of St. Margaret's Works, which will record the amount of atmospheric pollution which occurs in Leicester. The plan is to collect in large bottles, beneath a receiver, the rain which descends from the polluted atmosphere. The bottles are to be changed once a month for analysis. The report on the results of this experiment is awaited with interest.

